# A Colon-Targeted Prodrug, 4-Phenylbutyric Acid-Glutamic Acid Conjugate, Ameliorates 2,4-Dinitrobenzenesulfonic Acid-Induced Colitis in Rats

**DOI:** 10.3390/pharmaceutics12090843

**Published:** 2020-09-03

**Authors:** Soojin Kim, Seunghyun Lee, Hanju Lee, Sanghyun Ju, Sohee Park, Doyoung Kwon, Jin-Wook Yoo, In-Soo Yoon, Do Sik Min, Young-Suk Jung, Yunjin Jung

**Affiliations:** 1 College of Pharmacy, Pusan National University, Busan 46241, Korea; wldhel3507@hanmail.net (S.K.); shyun9122@naver.com (S.L.); purity4127@naver.com (H.L.); jsh141002@naver.com (S.J.); psh7728@pusan.ac.kr (S.P.); kwondoy@gmail.com (D.K.); jinwook@pusan.ac.kr (J.-W.Y.); insoo.yoon@pusan.ac.kr (I.-S.Y.); 2 College of Pharmacy, Yonsei University, 85 Songdogwahak-ro, Yeonsu-gu, Incheon 21983, Korea; minds@yonsei.ac.kr

**Keywords:** 4-phenylbutyric acid, colon-targeted drug delivery, colitis, prodrug, endoplasmic reticulum stress, chemical chaperone

## Abstract

An elevated level of endoplasmic reticulum (ER) stress is considered an aggravating factor for inflammatory bowel disease (IBD). To develop an ER-stress attenuator that is effective against colitis, 4-phenylbutyric acid (4-PBA), a chemical chaperone that alleviates ER stress, was conjugated with acidic amino acids to yield 4-PBA-glutamic acid (PBA-GA) and 4-PBA-aspartic acid (PBA-AA) conjugates. The PBA derivatives were converted to 4-PBA in the cecal contents, and the conversion was greater with PBA-GA than that with PBA-AA. After oral administration of PBA-GA (oral PBA-GA), up to 2.7 mM PBA was detected in the cecum, whereas 4-PBA was not detected in the blood, indicating that PBA-GA predominantly targeted the large intestine. In 2,4-dinitrobenzenesulfonic acid-induced colitis in rats, oral PBA-GA alleviated the damage and inflammation in the colon and substantially reduced the elevated levels of ER stress marker proteins in the inflamed colon. Moreover, PBA-GA was found to be as effective as the currently used anti-IBD drug, sulfasalazine. In conclusion, PBA-GA is a colon-targeted prodrug of 4-PBA and is effective against rat colitis probably via the attenuation of ER stress in the inflamed colon.

## 1. Introduction

Inflammatory bowel disease (IBD), which includes ulcerative colitis and Crohn’s disease, is an incurable and chronic disease of the gastrointestinal (GI) tract with multifaceted and challenging clinical manifestations. Although the pathogenesis and etiology of IBD remain to be fully elucidated, progress in the past two decades has enhanced our understanding of the immunological and genetic aspects of IBD. Whatever its fundamental pathological cause, the dysregulated host immune response caused by the gut microflora, as well as genetic and environmental factors, results in mucosal inflammation. This leads to a vicious cycle of inflammation–mucosal destruction–influx of antigens–immune response–inflammation, thereby perpetuating the unresolved inflammatory state [1,2].

The understanding of IBD pathogenesis, although obscure, has been translated to pharmacotherapy using drugs, such as aminosalicylates, glucocorticoids, immunosuppressants, and the burgeoning class of biologics, including anti-tumor necrosis factor-α agents [3,4]. The purpose of these IBD therapeutics is to induce the remission of destructive inflammation and maintain long-term remission. In addition to low efficacy (i.e., of aminosalicylates) and intolerable adverse effects, resistance to these therapies and the induction of tolerance pose great challenges to the treatment of IBD [5,6]. There is, therefore, an unmet medical need for the development of new IBD drugs with therapeutic and toxicological improvements.

The intestinal epithelium—the physical barrier between the mucosal layer and the gut luminal environment—plays a crucial role in mucosal homeostasis through the regulation of the immune response, composition of microflora, intestinal pathogens, and formation of the mucus layer, which is mediated by anti-microbial peptides, mucin, cytokines, chemokines, and hormones secreted from four types of intestinal epithelial cells, namely, Paneth cells, goblet cells, enteroendocrine cells, and absorptive enterocytes [7,8,9,10]. This role has been demonstrated in a number of animal- and genome-wide association studies wherein genes closely related to epithelial functions have been identified [11,12]. With the physiological importance of the intestinal epithelium for mucosal homeostasis, a growing body of evidence demonstrates that intestinal epithelial dysfunction is associated with the development of various gastrointestinal disorders, including IBD [13,14,15].

Recent studies have shown that cellular stress signaling, including endoplasmic reticulum (ER) stress signaling and the unfolded protein response (UPR), is linked to the normal function of multiple epithelial cell populations in the gut [16,17]. Accordingly, uncontrolled ER stress impairs mucosal barrier function, dysregulating the innate or adaptive immune response of the host cells and modulating the intestinal microbiota, which are established pathological conditions in IBD [16,17,18,19]. In fact, ER stress is associated with susceptibility to IBD, and signs of ER stress are commonly found in the ileal and colonic epithelia of patients with active IBD [17,20]. Therefore, interventions for restoring ER function in intestinal epithelial cells are considered therapeutic modalities in the treatment of IBD [21].

ER stress involves the increased production of misfolded proteins, leading to the UPR. Under controlled conditions, the activation of the UPR mitigates cellular ER stress via the elimination of misfolded proteins by preventing their aggregation and promoting degradation. If cells are subjected to excessive and continuous ER stress that is beyond the threshold that they can withstand, the cells undergo apoptosis. These cellular responses are mediated by three ER membrane proteins that sense stress; these proteins are protein kinase RNA-like ER kinase, inositol-requiring enzyme 1α, and activating transcription factor 6α, each of which is involved in signaling pathways that transduce cellular signals, culminating in homeostatic responses and apoptosis [22,23].

The aim of pharmacological interventions in ER stress is to facilitate the elimination of misfolded proteins, thereby preventing potentially harmful responses to uncontrolled ER stress, such as apoptosis and immune reactions, which would lead to the impairment of the mucosal barrier and inflammation [21,24]. Small molecules that facilitate the elimination of misfolded or unfolded proteins by binding to them and restoring ER function are defined as chemical chaperones [25,26,27]. 4-phenylbutyric acid (4-PBA), an FDA-approved drug for the treatment of urea cycle disorders, is a representative chemical chaperone. Owing to its safety and effectiveness against ER stress [28], the anti-colitic activity of 4-PBA has been investigated [29,30,31]. Consistent with the therapeutic hypothesis, 4-PBA reduces ER stress in the inflamed large intestine and shows considerable anti-colitic effects in animal models. However, its effective doses of 500 to 1000 mg/kg are too high for clinical application.

Colon-targeted drug delivery (CTDD) refers to the transport of an orally administered drug to the large intestine without a loss (absorption and metabolism) of the drug in the stomach and small intestine [32,33]. Therefore, in general, the colonic drug delivery results in the accumulation of greater concentrations of the drug at the target site (colon) than possible with that using conventional delivery, indicating that the dose of a drug required for the treatment of colonic diseases, such as IBD, can be reduced by CTDD. Moreover, CTDD tends to have a reduced risk of systemic side effects owing to the reduced dose and because the physiological features of the large intestine negatively influence drug absorption. Thus, this delivery technique is usually considered in the development of anti-colitic agents [32].

In this study, we tested whether the colon-targeted delivery of 4-PBA was effective against colitis, potentiated the anti-colitic activity of this drug, and could reduce the therapeutic dose to practically achievable levels. Colon-targeted prodrugs of 4-PBA were designed and synthesized and their colon-targeting property was evaluated. Furthermore, the therapeutic effects against colitis were assessed in a 2,4-dinitrobenzenesulfonic acid (DNBS)-induced model of colitis in rats.

## 2. Materials and Methods

### 2.1. Materials

4-PBA, 1,1′-carbonyldiimidazole (CDI), DNBS, L-glutamic acid dimethyl ester hydrochloride, L-aspartic acid dimethyl ester hydrochloride, and sulfasalazine (SSZ) were obtained from Tokyo Kasei Kogyo Co. (Tokyo, Japan). Phosphate-buffered saline (PBS, pH 7.4) was supplied by Thermo Fisher Scientific (Waltham, MA, USA). All organic solvents were purchased from Junsei Chemical Co. (Tokyo, Japan) and used in experiments without further purification. All other chemicals were commercially available products of reagent grade. A Varian FT-IR spectrophotometer (Varian, Palo Alto, CA, USA) and an AS 500 spectrometer (Varian) were used to record infrared (IR) spectra and ^1^H-NMR spectra, respectively. The chemical shifts in the NMR spectra were reported in ppm, downfield of tetramethylsilane. Elemental analysis was carried out by an Elemental Analyzer System (Profile HV-3, Manchester, UK). Mass analysis was performed using electrospray ionization mass spectrometry (Agilent QQQ 6460 mass spectrometer, Agilent Technologies, Santa Clara, CA, USA).

### 2.2. Synthesis of N-(4-Phenylbutanoyl) Glutamic Acid (PBA-GA) and N-(4-Phenylbutanoyl) Aspartic Acid (PBA-AA)

4-PBA (1.0 mmol) was reacted with CDI (1.3 mmol), dissolved in 10.0 mL of acetonitrile for 30 min, and L-glutamic acid dimethyl ester hydrochloride (2.0 mmol) was added. Then, the mixture was stirred at 20 °C for 24 h. The solvent in the reaction mixture was evaporated. The residue was dissolved in ethyl acetate (EA), washed thrice with 0.1 M HCl and 0.1 M NaOH, dried over anhydrous Na_2_SO_4_, and the residual solvent evaporated. The residue was dissolved in 8.0 mL of a 0.5 M NaOH solution and stirred at 40 °C for 30 min. The resulting solution was acidified using 1.0 M HCl solution and extracted using EA. The organic layer was dried over anhydrous Na_2_SO_4_ and the solvent was evaporated to obtain PBA-GA as an oil. PBA-AA was synthesized using L-aspartic acid dimethyl ester hydrochloride using the same method. The scheme for the synthesis of the 4-PBA derivatives is shown in Supplementary Figure S1 The formation of PBA-GA and PBA-AA was confirmed via IR and ^1^H-NMR. PBA-GA, yield: 67%; IR (nujol mull), ν_max_ (cm^−1^): 1713 (C = O, -COOH), 1628 (C = O, -CONH); ^1^H-NMR (DMSO-*d*6): 1.82–1.72 (m, 2H), 1.99–1.92 (m, 1H), 1.82–1.72 (m, 1H), 2.13 (t, J = 7.3 Hz, 2H), 2.32–2.23 (m, 2H), 2.56 (t, J = 7.7 Hz, 2H), 4.20 (td, J = 9.0, 5.1 Hz, 1H), 7.21–7.15 (m, 3H), 7.27 (t, J = 7.5 Hz, 2H); C_15_H_19_NO_5_ (293.31); calculated: C, 61.42; H, 6.53; N, 4.78; found: C, 61.12; H, 6.35; N, 4.69; C_15_H_19_NO_5_; m/z [M-H]^-^ calculated: 292.30; found: 292.64. PBA-AA, yield: 61%; IR (nujol mull), ν_max_ (cm^−1^): 1718 (C = O, -COOH), 1625 (C = O, -CONH); ^1^H-NMR (DMSO-*d*6): 1.82–1.73 (m, 2H), 2.12 (t, J = 7.3 Hz, 2H), 2.55 (dd, J = 14.5, 7.4 Hz, 2H), 2.69 (dd, J = 16.4, 5.6 Hz, 1H), 2.55 (dd, J = 14.5, 7.4 Hz, 1H), 4.53 (dd, J = 13.4, 7.7 Hz, 1H), 7.21–7.15 (m, 3H), 7.27 (t, J = 7.5 Hz, 2H). C_14_H_17_NO_5_ (279.29); calculated: C, 60.21; H, 6.13; N, 5.02; found: C, 60.54; H, 6.11; N, 5.05; C_14_H_17_NO_5_; m/z [M-H]^-^ calculated: 278.28; found: 278.47.

### 2.3. High-Performance Liquid Chromatography (HPLC)

The HPLC system consisted of a Gilson model 306 pump, 151 variable UV detector, and model 234 autoinjector (Gilson, Middleton, WI, USA). A Symmetry C18 column (HECTOR, RStech, Cheongju, Korea; 250 × 4.6 mm, 5 μm), with an attached guard column (Waters, 20 × 4.6 mm), was used for chromatographic separation. Samples from each experiment were filtered through membrane filters (0.45 μm). HPLC analyses of PBA, PBA-GA, and PBA-AA were conducted at a flow rate of 1.0 mL/min using mobile phases comprising methanol and 0.5% acetic acid (6.5:3.5, *v*/*v*). 5-chlorosalicylic acid was used as an internal standard (IS). The absorbance of the eluate was monitored at 214 nm using a UV detector, with a sensitivity of absorbance units full scale 0.01. The retention times of 4-PBA, PBA-GA, and PBA-AA were 12.4, 5.9, and 10.5 min, respectively. The HPLC method for 4-PBA analysis was validated as described previously [34]. Stock solutions of 4-PBA (1 mg/mL) were prepared by dissolving 10 mg of the drug in 10 mL of PBS (for plasma) or MeOH (for cecal contents). Calibration standards for plasma (1–50 μg/mL) were prepared by spiking the working standard solutions and IS into 300 μL of blank rat plasma. The total volume of the added solutions was adjusted to 25 μL with PBS. Calibration standards (for cecal contents) were prepared by spiking working standard solutions and IS into 100 μL of 10% of the cecal contents of normal rats. The final concentrations of the standard curve samples were 1–50 μg/mL. The IS concentration was 3.5 μg/mL in each sample. Quality control samples were prepared at three concentrations (1, 10, and 50 μg/mL). The detection limits were approximately 0.7 μg/mL (plasma) and 0.5 μg/mL (cecal contents) under our experimental conditions. Accuracy and relative standard deviations were 98.3% and 0.44% for plasma and 98.9% and 0.47% for cecal contents, respectively. A similar validation was performed for the analysis of PBA-GA in cecal contents. The detection limit was 0.9 μg/mL and accuracy and relative standard deviation were 97.9% and 0.49%, respectively. Gilson Trilution^®^LC software (v4.0, Middleton, WI, USA), was used for data analysis.

### 2.4. Apparent Distribution Coefficient and Chemical Stability

To 1.0 mM solutions of 4-PBA, PBA-GA, and PBA-AA prepared in 5.0 mL of 1-octanol pre-saturated with isotonic phosphate buffer (pH 6.8), an aqueous solution of 5.0 mL of isotonic phosphate buffer (pH 6.8) pre-saturated with 1-octanol was mixed. The mixture was shaken for 24 h and allowed to stand for 4 h for phase separation at 25 °C. The concentration of each compound in the aqueous phase was determined using a Shimadzu UV-Vis spectrophotometer (Tokyo, Japan) at 214 nm. The apparent distribution coefficients (*D_6.8_*) were calculated using the following equation:

(1) $$\log\;D_{6.8}=\log(C_{Oc}/C_{W})=\;\log\left[(C_{Oc}/(C_{O}-C_{Oc}\right)]$$

*C_O_*: initial concentration of compound in 1-octanol
*C_Oc_*: equilibrium concentration of compound in 1-octanol
*C_W_*: equilibrium concentration of compound in isotonic phosphate buffer (pH 6.8)

PBA-GA and PBA-AA (1.0 mM) were tested for chemical stability in HCl-NaCl buffer (pH 1.2) and isotonic phosphate buffer (pH 6.8). Each compound was incubated in these buffers for 24 h and the concentration was measured using HPLC.

### 2.5. Animals

Seven-week-old male Sprague-Dawley (SpD) rats were purchased from Samtako Bio Korea (Kyeong-gi-do, South Korea) and housed in the animal care facility at Pusan National University, Busan, South Korea, under controlled temperature, humidity, and dark/light cycle conditions. The animal protocol used in this study was reviewed and approved by the Pusan National University–Institutional Animal Care and Use Committee (PNU–IACUC) for ethical procedures and scientific care (approval no: PNU-2019-2392).

### 2.6. Incubation of PBA-GA and PBA-AA with the Contents of the Small and Large Intestines of the Rats

A male SpD rat (250–260 g) was sacrificed using CO_2_ euthanasia and a midline incision was made. The contents of the proximal small intestine, distal small intestine, and cecum were collected to prepare a 10% or 20% (*w*/*v*) suspension in isotonic phosphate buffer (pH 6.8). Drugs were incubated with cecal contents in a nitrogen gas bag (AtmosBag, Sigma). PBA-GA and PBA-AA (4.0 mL, 10 mM) dissolved in isotonic phosphate buffer (pH 6.8) were individually mixed with 4.0 mL of the 10% (*w*/*v*) suspension. The mixture was incubated at 37 °C. For PBA-GA, an additional experiment was performed using a 20% (*w*/*v*) suspension. At appropriate time points, samples were collected and centrifuged at 10,000× *g* for 5 min. Supernatants (0.1 mL) were mixed with methanol (0.9 mL) to remove proteins, followed by vortex mixing and centrifugation at 20,000 × *g* at 4 °C for 10 min. Lastly, supernatants were filtered through a membrane filter (0.45 μm) and the filtrate was subjected to HPLC to measure the concentration of each drug in the supernatants.

### 2.7. Determination of Drug Concentration in Blood and Cecum

Male SpD rats *had access* to *water* ad libitum during *fasting* for 24 h. 4-PBA (30.0 mg/kg) or PBA-GA (53.6 mg/kg, equivalent to 4-PBA 30.0 mg/kg) was dissolved in PBS (1.0 mL) and administered to rats by oral gavage. To measure 4-PBA concentration in the blood, blood samples were collected by cardiac puncture 0.25, 2, and 6 h after oral gavage. Blood samples were centrifuged at 10,000× *g* at 4 °C for 10 min to obtain the plasma (0.3 mL), which was mixed with 10% perchloric acid (75.0 μL) and 5-chlorosalicylic acid (25.0 μL, 1.0 mM) by vortex mixing, and then centrifuged at 10,000× *g* at 4 °C for 5 min. The supernatants (0.25 mL) were transferred to centrifuge tubes containing 7.6 μL of saturated K_2_CO_3_ solution and centrifuged for another 5 min [35]. To the supernatant (0.2 mL), 0.1 M HCl (0.6 mL) was added, which was followed by extraction with EA (0.7 mL). The organic layer (0.5 mL) was evaporated; the residue was dissolved in the mobile phase (0.15 mL) and filtered through a membrane filter (0.45 μm). The filtrate (20.0 μL) was subjected to HPLC.

To determine drug concentrations in the cecum, the contents of the cecum obtained 0.25, 2, and 6 h after oral gavage were mixed with isotonic phosphate buffer (pH 6.8) to make 10% suspensions. After centrifugation at 10,000× *g* for 10 min at 4 °C, methanol (0.9 mL) was added to 0.1 mL of the supernatant. The mixture was filtered through a membrane filter (0.45 μm) and subjected to HPLC. Five rats were used for each drug and time point.

### 2.8. DNBS-Induced Colitis in Rats

Experimental colitis was induced in rats as described previously [36,37]. Briefly, prior to colitis induction, male SpD rats (250–260 g) were starved for 24 h, except for access to water. Isoflurane (Hana Pharm, Hwaseong, Korea), supplied using a Small Animal O_2_ Single Flow Anesthesia System (LMS, Pyeongtaek, Korea), was used to induce colitis. Isoflurane concentration was 3% for induction and 2% for maintenance, with 1 L/min oxygen. When the rats no longer responded to physical stimuli under anesthesia, a rubber cannula (2.0 mm, OD) was inserted rectally into the colon such that the tip was 8 cm proximal to the anus. DNBS (48.0 mg), dissolved in 50% aqueous ethanol (0.4 mL), was instilled into the colon via the cannula.

### 2.9. Evaluation of the Anti-Colitic Effect

**Figure 1:**
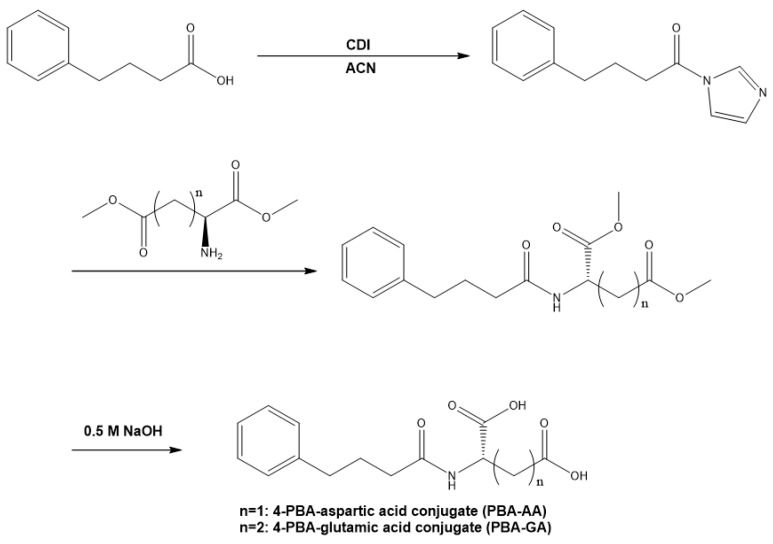
Scheme for the synthesis of *N*-(4-phenylbutanoyl) glutamic acid (PBA-GA) and *N*-(4-phenylbutanoyl) aspartic acid (PBA-AA). ACN: acetonitrile, CDI: 1,1′-carbonyldiimidazole.

To evaluate the anti-colitic effect of PBA-GA, two independent animal experiments were conducted. In the first experiment, rats were divided into five groups (*n* = 5 per group) and treated as follows: normal group: oral gavage of 1.0 mL of PBS; colitis control group: oral gavage of 1.0 mL of PBS; 4-PBA-treated colitis group: oral gavage of 4-PBA (30.0 mg/kg) in 1.0 mL of PBS; PBA-GA-treated colitis group L: oral gavage of PBA-GA (17.9 mg/kg, equivalent to 10.0 mg/kg of 4-PBA) in 1.0 mL of PBS; and PBA-GA-treated colitis group H: oral gavage of PBA-GA (53.6 mg/kg, equivalent to 30.0 mg/kg of PBA) in 1.0 mL of PBS. In the second experiment, rats were divided into four groups (*n* = 5 per group) and treated as follows: normal group: oral gavage of 1.0 mL of PBS; colitis control group: oral gavage of 1.0 mL of PBS; PBA-GA-treated colitis group: oral gavage of PBA-GA (53.6 mg/kg) in 1.0 mL of PBS; and SSZ-treated colitis group: oral gavage of SSZ (30.0 mg/kg) in 1.0 mL of PBS. After the induction of colitis for 3 days, each drug was administered to the colitic rats via oral gavage once daily for 7 days to evaluate the anti-colitic effects of the drugs. A scheme of the in vivo experiment is shown in Figure 1. A colonic damage score (CDS) was assigned to the colon of rats according to previously reported criteria [37,38]. The modified scoring system was as follows: 0, normal appearance; 1, localized hyperemia but no ulcer; 2, linear ulcers without significant inflammation; 3, 2–4 cm site of inflammation and ulceration; 4, serosal adhesion to other organs, 2–4 cm site of inflammation and ulceration; and 5, stricture, serosal adhesion involving several bowel loops, <4 cm site of inflammation, and ulceration. Four independent observers blinded to the treatment conditions performed the CDS assessment. The myeloperoxidase (MPO) activity in the distal colon (4.0 cm) was measured as described previously [37]. The distal colon samples were obtained by excising the distal part of the colon up to 4 cm from the rectum, and the lumens of the samples were gently irrigated with chilled PBS to remove the contents. The inflamed colon (0.2 g) was disrupted in a vial containing 2.0 mL of a 5.0% hexadecyltrimethylammonium bromide (pH 6.0) solution, homogenized, and incubated on ice for 20 min. The homogenate was centrifuged at 10,000× *g* at 4 °C for 10 min; 0.1 mL of the supernatant was mixed with 2.9 mL of 0.05 M phosphate buffer (pH 6.0) containing o-dianisidine (16.7 mg) and 30.0% H_2_O_2_ (1.7 mL), and MPO activity was measured using a UV spectrophotometer (Shimadzu, Tokyo, Japan) for 5 min at 460 nm. One unit of MPO activity was defined as the amount of enzyme that degraded 1.0 μmol of peroxide per minute at 25 °C.

### 2.10. Western Blot Analysis

To prepare lysates of the distal colon, tissue samples (0.2 g) were disrupted and homogenized in 2.0 mL of ice-cold radioimmunoprecipitation assay buffer (50 mM Tris-HCl (pH 7.4), 1 mM ethylenediaminetetraacetic acid 0.7% Na deoxycholate, 1% NP-40, 150 mM NaCl, 0.3 μM aprotinin, 1 μM pepstatin, and 1 mM phenylmethylsulfonyl fluoride or the ProEXTM CETi protein extraction solution (Translab, Daejeon, Korea) to determine the levels of ER stress marker proteins. After incubation on ice for 30 min, homogenates were centrifuged at 10,000× *g* at 4 °C for 20 min. The concentrations of proteins in the supernatants were determined using the bicinchoninic acid reagent (Thermo Fisher Scientific, Waltham, MA, USA) according to the manufacturer’s instructions. The tissue lysates were electrophoretically separated on 7.5%, 10.0%, or 15.0% sodium dodecyl sulfate–polyacrylamide gels and transferred onto nitrocellulose membranes (Bio-Rad, Hercules, CA, USA). The membrane was blocked with Tris-buffered saline with 0.1% Tween-20 (TBS-T), containing 5.0% skimmed milk powder, for 30 min at 25 °C. Cyclooxygenase (COX)-2, inducible nitric oxide synthase (iNOS), ATF6, and C/EBP homologous protein (CHOP) were detected using anti-COX-2 (sc-37,6861, Santa Cruz Biotechnology, Dallas, TX, USA), anti-iNOS (NOS-2) (sc-7271, Santa Cruz Biotechnology), anti-ATF6 (NBP1-40256, Novus Biologicals, Centennial, CO, USA), and anti-CHOP (L63F7, Cell Signaling Technology, Danvers, MA, USA) antibodies. The expression levels were normalized to those of α-tubulin or β-actin (Santa Cruz Biotechnology). After incubating the membranes with the appropriate secondary antibodies, they were detected using the EZ-Western Lumi Pico detection kit (DoGenBio, Seoul, Korea). Western blot images were quantified using Image Lab software (version 5.2 build 14, Bio-Rad, Hercules, CA, USA). The results are expressed as the means of quantified blots (*n* = 5).

### 2.11. Enzyme-Linked Immunosorbent Assay (ELISA) for Cytokine-Induced Neutrophil Chemoattractant-3 CINC-3)

To measure the levels of CINC-3 in the inflamed tissue, the distal colon was homogenized in potassium phosphate buffer (pH 6.0) and centrifuged at 10,000× *g* at 4 °C for 10 min. ELISA was performed using the CINC-3 ELISA kit (R&D Systems, Minneapolis, MN, USA) according to the manufacturer’s instructions.

### 2.12. Data Analysis

The results are expressed as the means ± standard deviation (SD). The differences between groups were tested using one-way analysis of variance (ANOVA) followed by Tukey’s HSD test or Mann–Whitney *U* test (for the CDS). Differences with α or *p* < 0.05 were considered statistically significant. SPSS 25 (IBM Corporation, New York City, NY, USA) was used for statistical analysis.

## 3. Results

### 3.1. Synthesis of 4-PBA Conjugated with Acidic Amino Acids

4-PBA was coupled with GA and AA to prepare the colon-targeted prodrugs, PBA-GA and PBA-AA. The chemical attachment of an amino acid as a carrier to a drug with a carboxylic functional group is a well-established approach in the design of colon-targeted prodrugs of specific drugs [32]. PBA-GA and PBA-AA were prepared via simple synthetic reactions as shown in Figure 1, and the formation of derivatives was verified using IR and ^1^H-NMR. In the IR spectra, the carbonyl stretching bands of the amide bonds formed by the conjugation of the amino acids with 4-PBA were observed at 1628 cm^−1^ and 1625 cm^−1^ for PBA-GA and PBA-AA, respectively. In the ^1^H-NMR spectra, proton signals originating from 4-PBA and the amino acids were detected along with a slight downfield shift of the signals. In addition, the α-carbon protons of the amino acids were downfield-shifted from 3.8 to 4.5, and amide protons ascribed to the formation of the amide bond were newly detected at approximately 8.1. The IR and ^1^H-NMR spectra of the conjugates and 4-PBA are shown in Supplementary Figure S2A,B.

### 3.2. Colon Specificity of PBA-AA and PBA-GA

**Figure 2:**
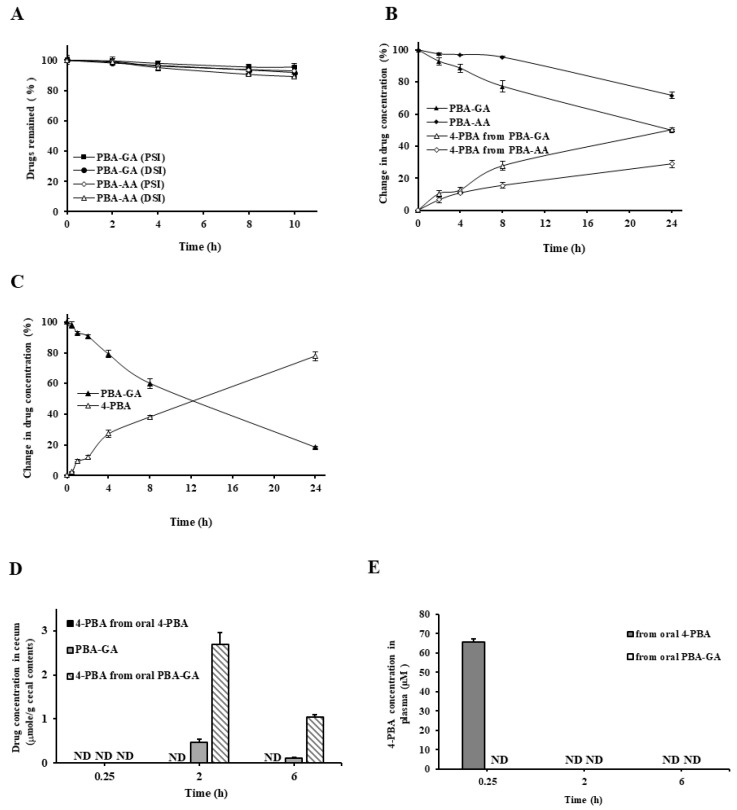
PBA-GA and PBA-AA are colon specific. (**A**) *N*-(4-phenylbutanoyl) glutamic acid (PBA-GA, 10.0 mM) and *N*-(4-phenylbutanoyl) aspartic acid (PBA-AA, 10.0 mM) were incubated with the contents of the proximal small intestine (PSI) and distal small intestine (DSI) suspended in phosphate-buffered saline (PBS; pH 6.8, 10.0%). (**B**) PBA-GA and PBA-AA were incubated with the cecal contents suspended in PBS (pH 6.8, 5.0%). (**C**) PBA-GA was incubated with the cecal contents suspended in PBS (pH 6.8, 10.0%). The levels of 4-PBA, PBA-GA, and PBA-AA were analyzed using HPLC. (**D**) Male SpD rats (250–260 g) were starved for 24 h and only provided water. 4-PBA (30.0 mg/kg) or PBA-GA (53.6 mg/kg, equivalent to 30.0 mg/kg of 4-PBA) suspended in PBS (pH 7.4) was administered to rats by oral gavage. The rats were sacrificed 0.25, 2, and 6 h after the oral gavage and the concentrations of 4-PBA and PBA-GA in the cecum were analyzed using HPLC. (E) Male SpD rats (250–260 g) were starved for 24 h and only provided water. 4-PBA (30.0 mg/kg) or PBA-GA (53.6 mg/kg, equivalent to 30.0 mg/kg of 4-PBA) suspended in PBS (pH 7.4) was administered to rats by oral gavage. Blood samples were obtained by cardiac puncture. 4-PBA levels were determined in the blood using HPLC. ND: not detectable. Five rats were used for each drug and time point in (**D**) and (**E**). The data in **A–E** represent the means ± SD (*n* = 5).

To examine the colon specificity of PBA-GA and PBA-AA, their DCs were determined. The *D_6.8_* of PBA-GA and PBA-AA were −1.56 and−1.86, respectively, and much lower than that of 4-PBA (2.53). These results suggest that the PBA-amino acid conjugates are more hydrophilic and less efficient than 4-PBA for passive transport through the epithelial layer of the GI tract. To verify the stability of PBA-GA and PBA-AA in the stomach and small intestine, their chemical stability was tested in buffers of pH 1.2 and 6.8. The concentrations of the drugs in both buffers did not change over 24 h. Next, PBA-GA and PBA-AA were incubated with the contents of the small intestine and cecum of rats and the concentrations of the conjugates and 4-PBA were measured at appropriate time points. Both amino acid conjugates disappeared, releasing 4-PBA into the contents of the cecum, but remained stable in the contents of the small intestine. In the cecal content, the disappearance rate of PBA-GA was greater than that of PBA-AA; thus, PBA-GA was used in subsequent in vivo experiments (Figure 2A,B). In the 10% cecal suspension, the disappearance of PBA-GA and the consequent release of 4-PBA was faster (Figure 2C). When a similar experiment was conducted with the autoclaved 10% cecal content [39], no release of 4-PBA was observed. These results suggest that the amino acid conjugates of 4-PBA are poorly absorbed and stable in the small intestine, whereas they are converted to 4-PBA by microbial enzymes in the large intestine.

To ensure that PBA-GA could efficiently deliver 4-PBA to the large intestine, the concentration of 4-PBA in the cecal content was measured at 0.25, 2, and 6 h after the oral gavage of PBA-GA (oral PBA-GA, equivalent to 30.0 mg/kg of 4-PBA) and 4-PBA (oral 4-PBA, 30.0 mg/kg). Both 4-PBA and PBA-GA were detected in the cecum when PBA-GA was administered orally, whereas no 4-PBA was detectable in the case of orally administered 4-PBA (Figure 2D). The maximum 4-PBA concentration in the cecum was approximately 2.7 mM after 2 h of the oral administration of PBA-GA. In agreement with the in vitro results for the cecal content, the concentration of PBA-GA was much lower than that of 4-PBA at each time point. To examine whether the colonic delivery of 4-PBA limited the systemic absorption of 4-PBA, 4-PBA (30.0 mg/kg) and PBA-GA (equivalent to 30.0 mg/kg of 4-PBA) were administered orally to rats, and the concentration of 4-PBA was monitored in the plasma after 0.25, 2, and 6 h. Up to approximately 65 μM 4-PBA was observed after oral administration of 4-PBA, whereas it was not detected after oral administration of PBA-GA, indicating that PBA-GA restricts the systemic absorption of 4-PBA (Figure 2E).

### 3.3. PBA-GA Mitigates DNBS-Induced Colitis in Rats

**Figure 3:**
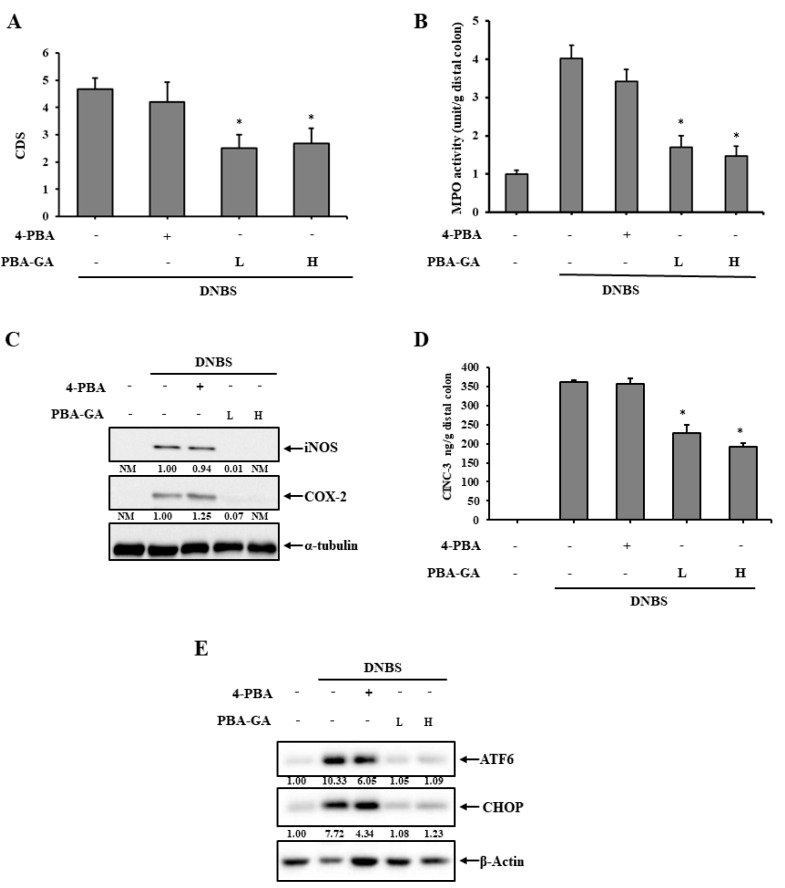
PBA-GA ameliorates 2,4-dinitrobenzenesulfonic acid (DNBS)-induced rat colitis by attenuating endoplasmic reticulum (ER) stress.(**A**–**E**) Three days after the induction of colitis, PBA-GA (17.9 (L) and 53.6 mg/kg (**H**), equivalent to 10 and 30.0 mg/kg of 4-PBA) dissolved in 1.0 mL of PBS (pH 7.4) was administered orally to rats once daily. The rats were sacrificed after the seventh dose. For comparison, 4-PBA (30.0 mg/kg) was administered in the same manner as PBA-GA. (**A**) The colonic damage score (CDS) was determined for each group, as described in the “Materials and Methods” section. (**B**) Myeloperoxidase (MPO) activity was measured in the inflamed distal colons (4.0 cm). Inflammatory mediators, including (**C**) iNOS, COX-2 and (**D**) CINC-3, were measured in the inflamed colon. α-Tubulin was used as a loading control to normalize the levels of iNOS and COX-2. (**E**) The levels of the ER stress marker proteins, ATF6 and CHOP, were monitored in the inflamed colon. β-Actin was used as a loading control to normalize the levels of ATF6 and CHOP. ^*^ *p* < 0.05 vs. DNBS control, NM: not measurable. The data represent the means ± SD (*n* = 5).

PBA-GA delivered millimolar concentrations of 4-PBA in the large intestine and 4-PBA (1-5 mM) effectively attenuates ER stress in cells [40,41]. Thus, whether PBA-GA mitigated colonic inflammation by preventing the induction of ER stress was examined. DNBS was instilled into the distal colon of the rats through the rectal route. Three days after the induction of inflammation, PBA-GA was orally administered to the colitic rats once daily for 7 days. A similar experiment was repeated with 4-PBA to examine whether the anti-colitic effects of PBA-GA were associated with the colonic delivery of 4-PBA. The dose of 4-PBA was 30.0 mg/kg, whereas PBA-GA was administered at two doses equivalent to 10.0 and 30.0 mg/kg of 4-PBA. Seven days after the administration of the drugs, rats were sacrificed and the anti-colitic activity was evaluated. DNBS induced severe inflammation and mucosal damage along with tissue edema, stenosis, and adhesion to the neighboring organs (Figure 3A and Figure S3). The oral administration of PBA-GA substantially mitigated the colonic damage at both doses, and there was no significant difference between the doses. In line with these findings, the MPO activity in the inflamed colonic tissues decreased from 4.3 to 1.7 units at 10.0 mg/kg and 1.4 units at 30.0 mg/kg of PBA-GA (Figure 3B). The molecular indices were also measured for the distal colon. PBA-GA decreased the production of inflammatory mediators, such as COX-2, iNOS (Figure 3C), and CINC-3 (Figure 3D), which were substantially increased in the inflamed colon tissue (Figure 3C,D). As for the macroscopic indices, no significant differences in the molecular effects were observed between the doses of PBA-GA. In contrast, no significant macroscopic and molecular effects against colitis were observed with oral 4-PBA. To confirm the relevance of the prevention of ER stress to the anti-colitic effects of PBA-GA, the levels of CHOP and ATF6 were determined in the inflamed distal colons. Colitis induced by DNBS markedly elevated the levels of the ER stress marker proteins. Although oral PBA-GA reduced the levels of these marker proteins to normal levels, a decrease of approximately 40% was observed with orally administered 4-PBA (Figure 3E). In accordance with the anti-colitic effects observed at the two doses of oral PBA-GA, no significant difference was observed in the reduction of the marker proteins at the two doses.

### 3.4. PBA-GA Is as Effective as SSZ in Mitigating DNBS-Induced Rat Colitis

**Figure 4:**
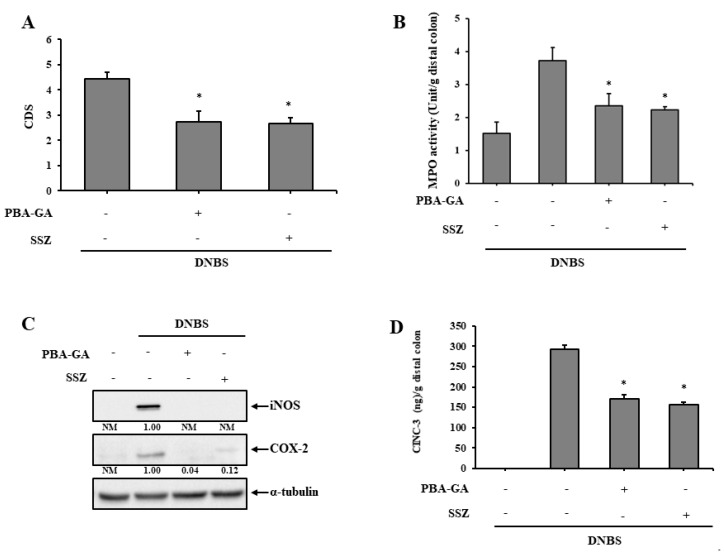
PBA-GA is as effective as sulfasalazine (SSZ) in ameliorating DNBS-induced rat colitis.(**A**–**D**) Three days after the induction of colitis, PBA-GA (53.6 mg/kg, equivalent to 30.0 mg/kg of 4-PBA) dissolved in 1.0 mL of PBS and SSZ (30.0 mg/kg) suspended in 1.0 mL PBS (pH 7.4) were administered orally to rats once daily. The rats were sacrificed after the seventh dose. (**A**) The colonic damage score (CDS) was determined for each group as described in the “Materials and Methods” section. (**B**) Myeloperoxidase (MPO) activity was measured in the inflamed distal colons (4.0 cm). Levels of inflammatory mediators, including (**C**) iNOS, COX-2 and (**D**) CINC-3, were measured in the inflamed colon. α-Tubulin was used as a loading control to normalize the levels of iNOS and COX-2. The data represent the means ± SD (*n* = 5). ^*^ *p <* 0.05 vs. DNBS control, NM: not measurable.

SSZ, a colon-targeted prodrug of 5-aminosalicylic acid (5-ASA), is an anti-inflammatory drug used clinically for the treatment of mild to moderate IBD. Thus, the anti-colitic effects of PBA-GA in the rat colitis model were compared to those of SSZ. SSZ (30.0 mg/kg) and PBA-GA (equivalent to 30.0 mg/kg of 4-PBA) were orally administered to the colitic rats once daily for 7 days. PBA-GA and SSZ reversed colonic damage and decreased MPO activity in the inflamed colonic tissues. PBA-GA was as effective as SSZ (Figure 4A,B and Figure S4). Moreover, the levels of inflammatory mediators, COX-2, iNOS, and CINC-3, were diminished in the inflamed colonic tissues with oral PBA-GA, and there was no significant difference in the anti-inflammatory effects between PBA-GA and SSZ (Figure 4C,D).

## 4. Discussions

In this study, we tested whether the colonic delivery of the chemical chaperone, 4-PBA, was effective against rat colitis and could be a feasible translatable treatment strategy. Despite the safety of this FDA-approved drug and its anti-colitic activity, the clinical use of 4-PBA for the treatment of gut inflammation might be limited owing to its very high effective dose (500–1000 mg/kg). To circumvent this obstacle, CTDD was used to deliver an orally active colon-targeted prodrug of 4-PBA that could be effective at a practical dose. The conjugation of acidic amino acids with drugs containing a carboxylic group is a well-established strategy in the design of colon-specific prodrugs. Thus, 4-PBA was conjugated with acidic amino acids, GA and AA, to yield PBA-GA and PBA-AA. As predicted, the 4-PBA derivatives exhibited colon specificity in vitro and in vivo and PBA-GA was superior to PBA-AA in colonic activation. As this would likely lead to greater availability of 4-PBA in the large intestine, which is more suitable for this study as it reduces the dose required, PBA-GA was used for subsequent in vivo experiments.

Consistent with the general advantages of CTDD [32], PBA-GA is likely to reduce the risk of systemic side effects owing to the absorption of 4-PBA during long-term treatment [42,43]. Oral PBA-GA did not afford 4-PBA to the blood, presumably by effectively preventing the absorption of 4-PBA. In contrast, up to 65.0 μM 4-PBA was detected in the blood after 15 min, although this then became undetectable 2 h after the oral administration of 4-PBA, showing rapid systemic absorption and metabolism, as previously reported [35]. In agreement with this observation, no 4-PBA was detected in the cecum of rats after the oral administration of 4-PBA (30.0 mg/kg), whereas up to 2.7 mM (0.44 mg/g cecal contents) 4-PBA was detected when PBA-GA (equivalent to 30.0 mg/kg of 4-PBA) was administered orally, further confirming the colon specificity of PBA-GA. Overall, a greater concentration of 4-PBA was observed in the large intestine than in blood after the administration of PBA-GA. This is a general benefit of CTDD that makes it possible to reduce the dose of 4-PBA in the treatment of colonic inflammation [32]. In addition, systemically absorbed 4-PBA is rapidly metabolized to 4-phenylacetate, mainly in the liver and kidney, thereby necessitating an increase in dose to maintain active 4-PBA levels in blood [35].

In agreement with these findings, although oral 4-PBA was not effective against rat colitis, oral PBA-GA effectively mitigated colonic damage and inflammation in rat colitis even at one-third the dose of 4-PBA. Oral PBA-GA at 53.6 mg/kg (equivalent to 30 mg/kg of 4-PBA) was slightly more effective than oral PBA-GA at 17.9 mg/kg (equivalent to 10 mg/kg of 4-PBA), but this difference was not significant. Since the SSZ dose required for the treatment of ulcerative colitis is between 1000 and 4000 mg/day in human adults [44], the use of these two doses of PBA-GA would not be impractical.

4-PBA is a chemical chaperone that reduces ER stress, which could be the potential pharmacological mechanism underlying the anti-colitic activity of 4-PBA. The oral administration of PBA-GA at a high dose resulted in the delivery and accumulation of millimolar concentrations of 4-PBA in the cecum; at these concentrations, 4-PBA significantly reduces the levels of the ER stress in various types of cells [40,41]. Moreover, oral PBA-GA administration decreased the elevated levels of the ER stress marker proteins in the inflamed colons to their normal levels. These findings may explain why such high doses of 4-PBA (500–1000 mg/kg) are needed to show efficacy in murine colitis [29,31], given that the elimination half-life is very short owing to rapid metabolism, although the oral administration of 5000 mg of 4-PBA affords concentrations of 1.18–1.32 mM 4-PBA in blood in humans [45]. Oral PBA-GA administration not only provides higher 4-PBA concentrations at the inflamed site but also avoids such rapid metabolism, thereby maintaining active therapeutic concentrations in the inflamed site for longer periods, even at much lower doses.

The oral administration of 4-PBA reduced the levels of the ER stress marker proteins to some degree, although it was not significantly effective against colitis in rats. These findings suggest that to exert anti-colitic activity, a greater reduction in ER stress than that provided by oral 4-PBA is required. The oral administration of PBA-GA reduced the levels of the ER stress markers to almost normal levels and was substantially effective against rat colitis. However, the manner in which oral 4-PBA reduces the levels of the ER stress marker proteins is not clear, as the blood concentration of 4-PBA obtained after oral 4-PBA administration may not be sufficient to decrease the levels of these proteins, as shown in our cell experiment (Figure 3A). Although, for now, no suitable explanation is available, it is possible that the 4-PBA-mediated reduction of ER stress might be cell-type specific. Other than colon epithelial cells, other types of cells in the mucosal layers, such as immune cells, may be more sensitive to 4-PBA; however, this theory needs to be tested using immune cells isolated from the colonic mucosal layer.

In the DNBS-induced rat colitis model, PBA-GA was observed to be as effective against colitis as SSZ. Unlike the suppression of inflammatory signals, such as nuclear factor (NF)-ĸB [46], by SSZ, PBA-GA is considered to exert its anti-colitic effects by protecting and fortifying the epithelial barrier. In addition, long-term NF-ĸB inhibition may impair epithelial integrity [47,48]. Thus, the combination of PBA-GA with a conventional drug may provide greater therapeutic benefit than that obtained with the use of such drugs alone. PBA-GA may reduce the risk of SSZ toxicity and the distinct anti-colitic mechanisms may enable combination therapy to elicit synergistic effects against colitis. For this reason, it is worth developing a colon-targeted mutual prodrug comprising 4-PBA and 5-ASA, which would be more beneficial and patient-friendly than the physical combination of SSZ and PBA-GA.

In conclusion, PBA-GA, a colon-targeted prodrug of the chemical chaperone 4-PBA, effectively mitigated colonic damage and inflammation in a DNBS-induced rat colitis model without significant systemic absorption of the drug and was as effective as SSZ. Moreover, the anti-colitic activity of PBA-GA was observed at a lower dose than that achievable with 4-PBA. Collectively, these results suggest that the colonic delivery of 4-PBA using PBA-GA is a feasible strategy to reduce the effective dose and potential side effects of 4-PBA and could improve patient compliance.

## Supplementary Materials

The following are available online at https://www.mdpi.com/1999-4923/12/9/843/s1. Figure S1: A scheme of the in vivo experiment. Figure S2A and B: Instrumental analysis of PBA-GA and PBA-AA. Figure S3: Photos of the serosal and luminal sides of the colons. Figure S4: Photos of the serosal and luminal sides of the colons.
