# Supplementary figures and images for: A Colon-Targeted Prodrug, 4-Phenylbutyric Acid-Glutamic Acid Conjugate, Ameliorates 2,4-Dinitrobenzenesulfonic Acid-Induced Colitis in Rats
Source: Pharmaceutics. 2020 Sep 3;12(9):843. doi: 10.3390/pharmaceutics12090843 (PMC7558321; doi:10.3390/pharmaceutics12090843)

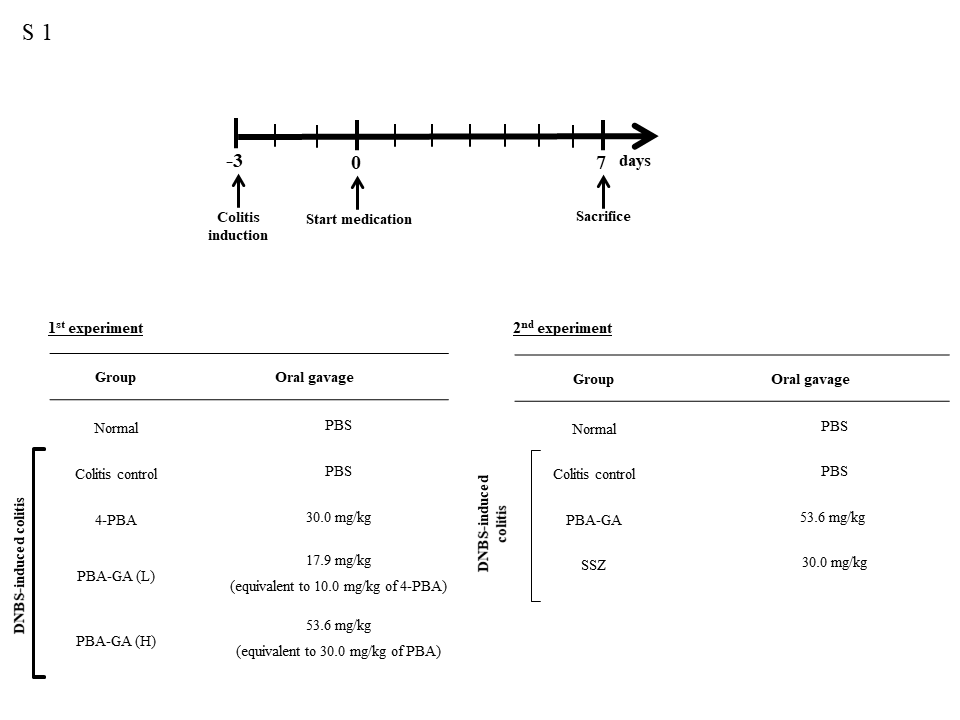

Supplement: Supplementary file 1 [file pharmaceutics-12-00843-s001.zip › S1.tif]

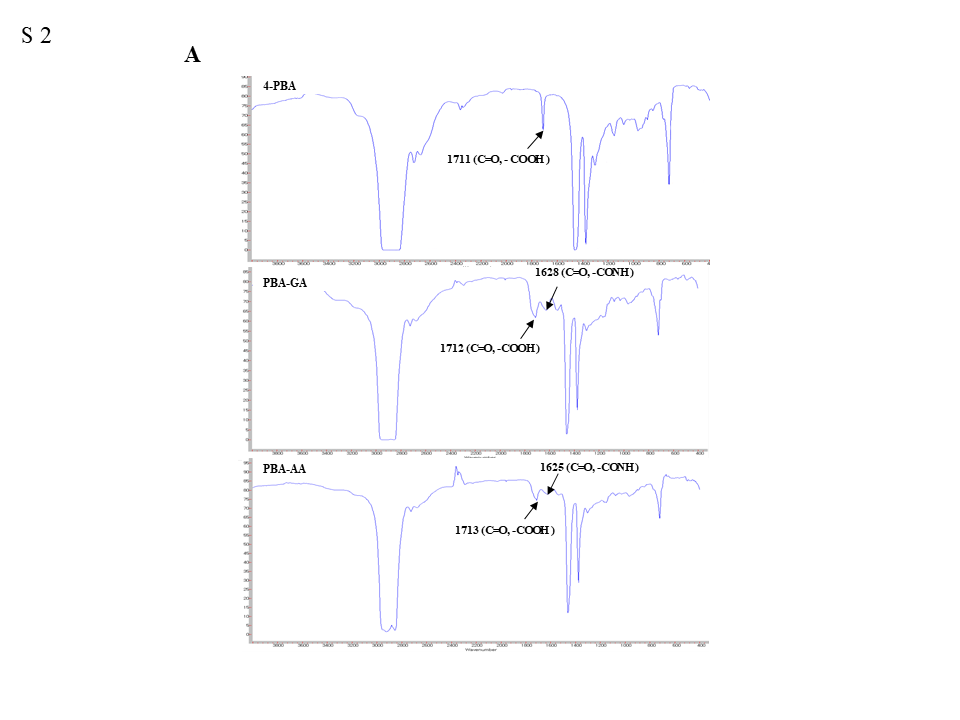

Supplement: Supplementary file 1 [file pharmaceutics-12-00843-s001.zip › S2-1.tif]

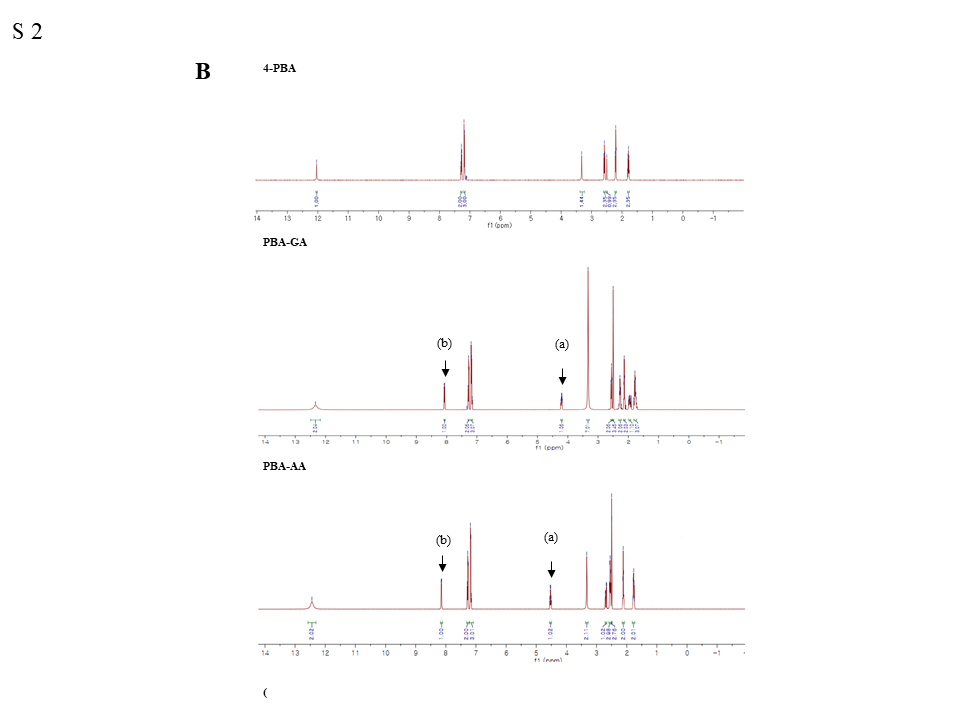

Supplement: Supplementary file 1 [file pharmaceutics-12-00843-s001.zip › S2-2.tif]

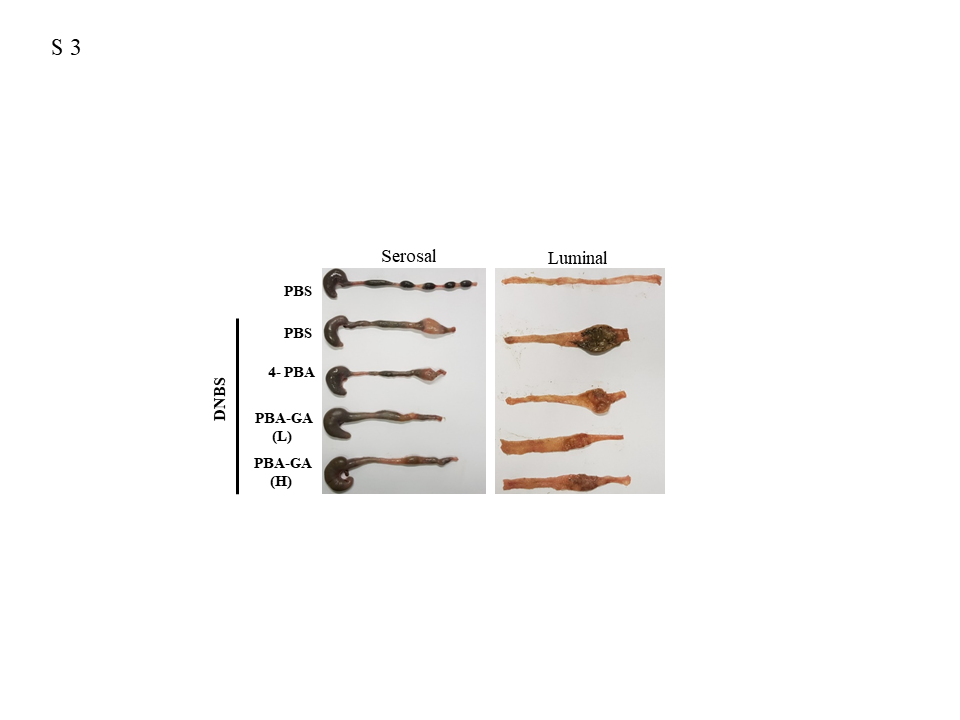

Supplement: Supplementary file 1 [file pharmaceutics-12-00843-s001.zip › S3.tif]

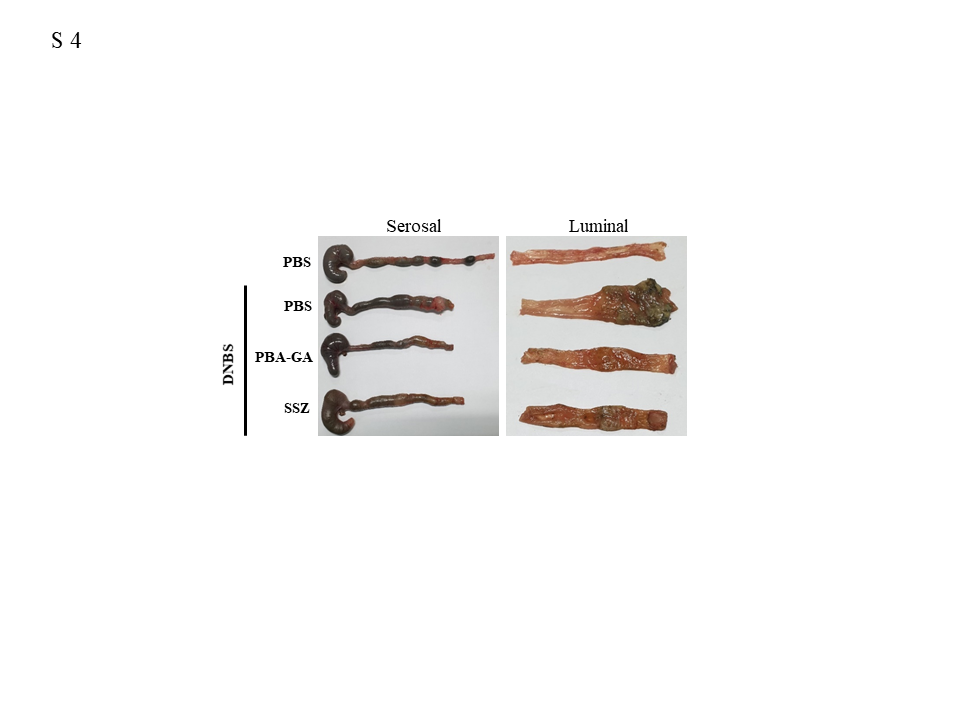

Supplement: Supplementary file 1 [file pharmaceutics-12-00843-s001.zip › S4.tif]
